# Application of contrast-enhanced ultrasound for extradigital glomus tumor in the thigh: a case report

**DOI:** 10.3389/fonc.2025.1645052

**Published:** 2025-09-04

**Authors:** Guangyuan Dai, Jiawen Fang, Shengmin Zhang

**Affiliations:** ^1^ Department of Ultrasound Medicine, The First Affiliated Hospital of Ningbo University, Ningbo, China; ^2^ Department of General Practice, Baiyun Community Health Service Center, Ningbo, China

**Keywords:** extradigital glomus tumors (EGTs), ultrasound, contrast-enhanced ultrasound, imaging manifestations, case report

## Abstract

Glomus tumors (GTs) are rare neoplasms, classically presenting as subungual lesions with pathognomonic triad of severe paroxysmal pain triggered by cold, pressure, or touch. Extradigital glomus tumors (EGTs) pose significant diagnostic challenges due to their anatomical atypicality and frequently ambiguous clinical manifestations. Ultrasonography remains the primary imaging modality for evaluating soft tissue masses. In the diagnostic assessment of GTs by sonographers, the identification predominantly relies on characteristic clinical presentations combined with the demonstration of intratumoral hypervascularity through color Doppler imaging, as grayscale ultrasound features exhibit nonspecific characteristics. However, conventional ultrasonography encounters significant diagnostic challenges in EGTs with atypical clinical presentations and deep-seated locations, primarily due to the markedly reduced sensitivity of color Doppler in assessing blood flow within deep soft tissue lesions. In contrast, contrast-enhanced ultrasound (CEUS) allows dynamic visualization of microvascular perfusion patterns, enabling precise evaluation of intratumoral hemodynamic characteristics. This technique may assist in enhancing diagnostic accuracy and guiding targeted biopsies. We report a case of a 56-year-old man with a 4-year history of drug-resistant left thigh pain. CEUS revealed a 21-mm intramuscular mass exhibiting rapid heterogeneous enhancement followed by rapid washout, suggesting hypervascularity. Histopathology confirmed a glomus tumor of uncertain malignant potential. This case highlights the clinical and imaging characteristics of EGTs, providing clinicians with a comprehensive understanding of this rare entity.

## 1 Introduction

Glomus tumors (GTs) are vascular structures composed of cells similar to normal smooth muscle cells, accounting for only 1% to 5% of soft tissue masses. They commonly occur at the arteriovenous anastomoses in the extremities, with 50% to 75% of cases found subungually in the hand (1). The usual clinical presentations consist of pain, tenderness at the site of the tumor, and hypersensitivity to cold (2, 3).Extradigital glomus tumors (EGTs) are even rarer, with localizations occurring in the following order of decreasing frequency: upper extremities, lower extremities, trunk, and face (4). Clinicopathologically, GTs are classified into three categories: benign glomus tumors, glomus tumors of uncertain malignant potential (GT-UMP), and malignant glomus tumors (3).

Given the perception that GTs are confined to the digits, accurate diagnosis of an EGT can be significantly delayed, leading to prolonged pain and suffering (5). Ultrasound and magnetic resonance imaging (MRI) can be performed when assessing whether a lesion is a GT; both have a similarly high sensitivity. However, MRI only has a specificity of about 50%, is more time-consuming to perform, and is more expensive (6). Ultrasound is a non-invasive, readily available, and cost-effective imaging modality that provides high-resolution images of soft tissue structures. Its high spatial resolution enables detailed assessment of tumor dimensions, anatomical relationships, and vascular architecture through Doppler interrogation - all critical elements for lesion characterization and surgical guidance. Previous reports have documented cases of EGTs; however, to the best of our knowledge, there are currently no case reports specifically describing the contrast-enhanced ultrasound (CEUS) features of EGTs. Herein, we report the CEUS findings of a patient with a EGT in the left thigh.

## 2 Case presentation

A 56-year-old male presented to our pain management clinic with a 4-year history of sharp, intermittent left thigh pain radiating to the lumbar region, demonstrating positional aggravation. There was no relationship between cold temperature exposure and his pain. Despite prolonged conservative management including pharmacotherapy and physical rehabilitation, progressive symptom escalation prompted specialized evaluation. Physical examination showed atrophy of the left thigh compared to the contralateral side, severe tenderness on palpation, no palpable mass, and no signs of infection or skin changes. The patient’s laboratory tests showed no significant abnormalities.

**Figure 1:**
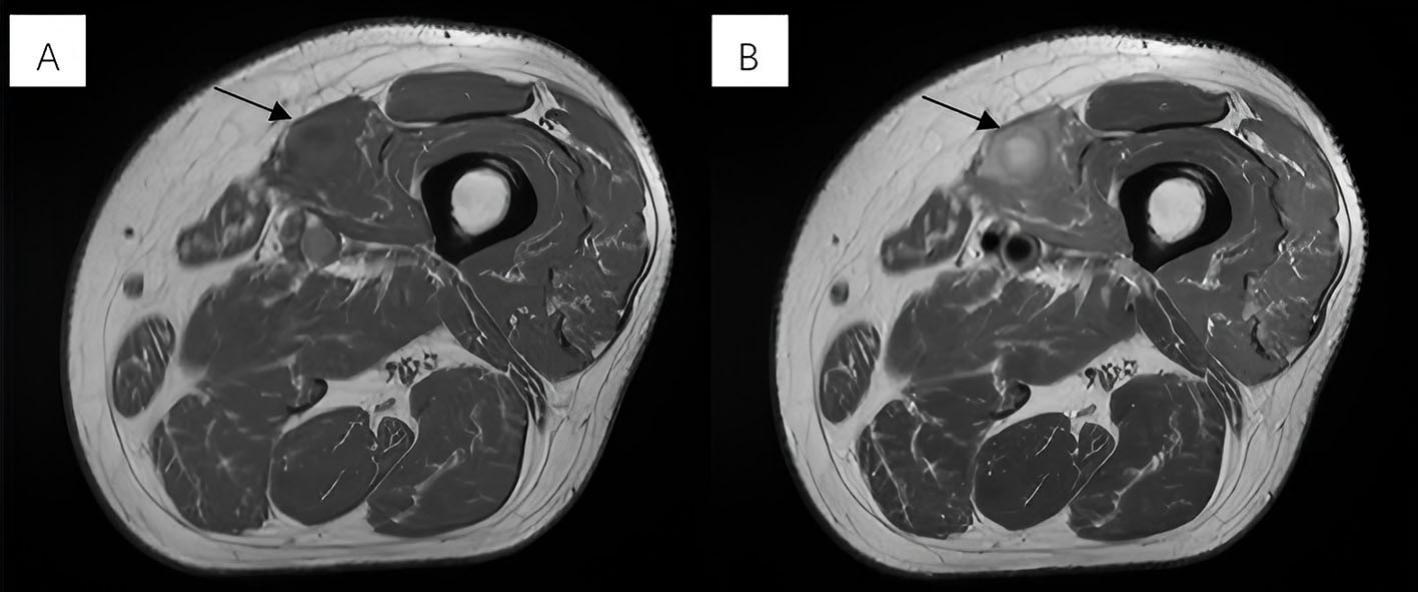
**(A)** Exhibiting iso-intensity on T1-weighted images. **(B)** Mixed hyperintensity on T2-weighted images.

For more evaluation, MRI revealed a near-oval lesion within the vastus medialis muscle with the largest diameter measuring approximately 25 mm. Exhibiting iso-intensity on T1-weighted images and mixed hyperintensity on T2-weighted images (**Figures 1A, B**). The lesion demonstrated significant hyperintensity on diffusion-weighted imaging (DWI). The MRI report suggested a tumor-like lesion within the left vastus medialis muscle, surgical intervention is recommended.

**Figure 2:**
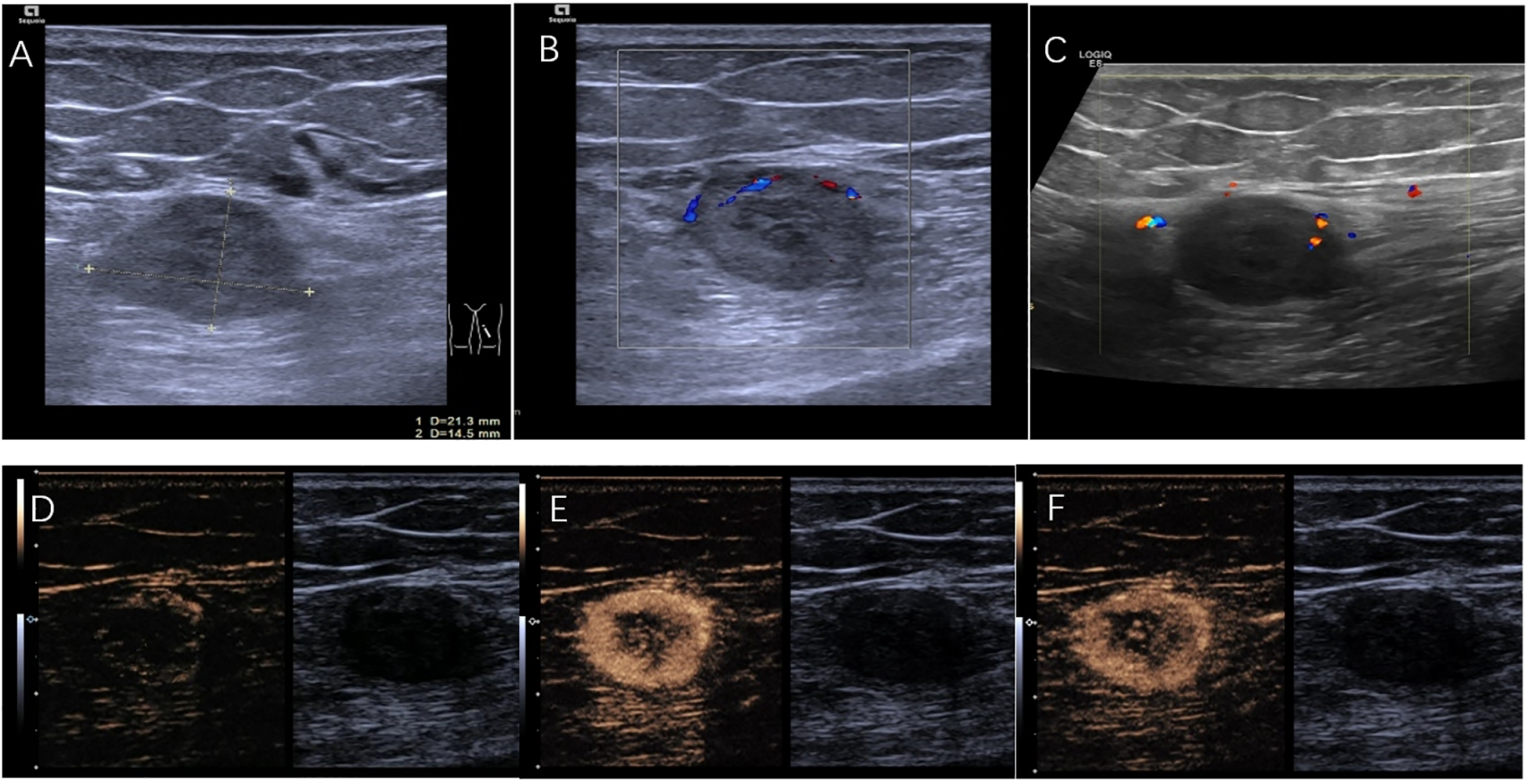
**(A)** A hypoechoic lesion of 21mm×14mm. **(B)** Color doppler performed with ACUSON Sequoia (linear array transducer operating at 18MHz; PRF:700 Hz; Wall Filter:60 Hz). **(C)** Color doppler performed with LOGIQ E8 (linear array transducer operating at 11MHz; PRF:800 Hz; Wall Filter:50 Hz). **(D)** CEUS demonstrates the lesion starting to enhance at the starting at 17s. **(E)** CEUS shows the lesion rapidly reaching its peak enhancement at 25 s. **(F)** CEUS illustrates the lesion fading at 32s.

The patient was referred to the ultrasound interventional department for ultrasound-guided percutaneous biopsy to establish a diagnosis. Preoperative ultrasound was performed, two-dimensional ultrasound imaging revealed an ovoid, solid, heterogeneous, hypoechoic mass located within the muscular layer of the left upper thigh, with 21×14 mm in size (**Figure 2A**). The discrepancy in lesion size between MRI and ultrasound measurements can be attributed to two factors: MRI measured the maximum single-axis diameter, while ultrasound documented orthogonal dimensions; slight measurement differences among radiologists due to subjective image interpretation thresholds. Color Doppler ultrasound evaluations were performed using two different ultrasound systems: the ACUSON Sequoia (Siemens Healthineers, Germany) equipped with a linear array transducer operating at 18MHz, and the LOGIQ E8 (GE Healthcare, USA) equipped with a linear array transducer operating at 11 MHz. Doppler parameters were optimized for deep tissue assessment: the Pulse Repetition Frequency (PRF) was set to a low value to enhance sensitivity to low flow velocities, the wall filter was minimized, the color gain was adjusted to just below the noise threshold, and the color box was focused over the lesion. Both systems consistently revealed only focal and linear blood flow signals within the tumor (**Figures 2B, C**). In further CEUS examination, SonoVue 2.4mL was injected into the median vein of the left upper limb, CEUS findings showed rapid enhancement of the lesion starting at 17s (**Figure 2D**), reaching a peak at 25s with rapid heterogeneous high enhancement (**Figure 2E**). There was no significant increase in the size of the mass after contrast enhancement compared to its size observed on grayscale ultrasound. Clearance began at 32s and rapid faded, observed up to 5 min (**Figure 2F**). CEUS suggested rapid enhancement and swift washout of the left thigh lesion.

**Figure 3:**
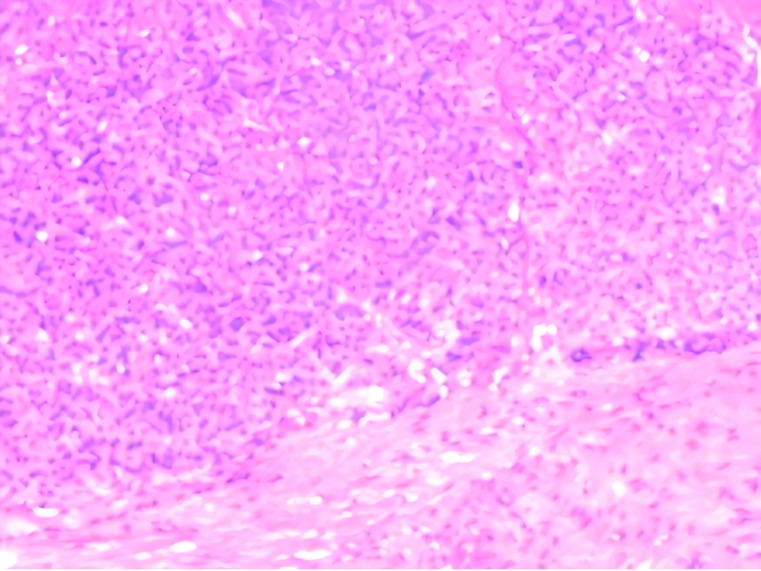
Histological examination at ×100 objective (H&E stain) showing monotonous small round cells arranged in sheets surrounding vascular channels. The tumor cells demonstrated pale eosinophilic cytoplasm with well-defined cell borders, containing centrally located round nuclei exhibiting homogeneous chromatin patterns. Mitotic figures were conspicuously absent.

Thereafter, an ultrasound-guided biopsy of the mass was performed. Histopathology revealed that the tumor cells exhibited uniform morphology, with weakly eosinophilic cytoplasm and rich vasculature. No evidence of mitotic figure or necrosis was seen. Immunohistochemistry showed positivity with SMA and Ki-67(<5%), vessel positivity with CD31 and CD34, while negative for S-100, Melanoma, CgA or TFE3. These findings were compatible with the diagnosis of GT(**Figure 3**). Due to the mass being located within the striated muscle, the pathological diagnosis was GT of uncertain malignant potential.

After that, the patient was scheduled for surgical excision. Under general anesthesia, the patient was positioned in the supine position on the operating table. A longitudinal skin incision was made along the biopsy entry point. This was followed by dissection of subcutaneous tissues focused and directed toward the biopsy tract until the lesion was identified within the vastus medialis muscle. Finally, a marginal excision of the mass was performed. Histopathological confirmation following surgical intervention corroborated the diagnostic accuracy of ultrasound-guided core needle biopsy. Postoperative recovery was uneventful. Sutures were removed at 2 weeks with complete wound healing. At the 3-week follow-up, the patient reported complete resolution of pain and resumed normal activities.

## 3 Discussion

GT is a rare type of vascular tumor that originates from the glomus bodies (7). Glomus bodies are neuro-myo-arterial structures located at the dermal-hypodermal junction. Their primary function is to control blood flow to the skin in response to variations in body temperature (8). GTs are most commonly found in the subungual regions of the fingers and toes, where glomus bodies are densely concentrated (9). These tumors typically present with sharp pain, point tenderness, and extreme sensitivity to cold (10). Although GTs typically occur in the digits, they can also arise anywhere in the skin and soft tissue. EGTs present greater diagnostic challenges due to atypical manifestations (7, 11). This diagnostic difficulty is reflected in Lee et al.’s finding of a 20% correct diagnosis rate for EGTs by initial physicians (11). Differential diagnosis of GTs includes other soft tissue tumors such as lipoma, neurofibroma, schwannoma, and hemangioma (12).

Histologically, the GT is typically a highly vascularized neoplasm with uniform rounded cells encircling capillary-sized vessels. These cells are round to polyhedral, containing granular eosinophilic cytoplasm and the nuclei have finely dispersed chromatin. The cells lack nuclear atypia and mitotic activity is rare (10). Glomus cells are immunohistochemically relatively non-specific, but show positivity for smooth muscle actin, vimentin and neuron specific enolase. They are usually negative for other markers of soft tissue tumors such as CD34, cytokeratin or S-100 (13, 14).

Most GTs are benign, while malignant GTs are rare and highly aggressive (15). Cases that do not completely fulfill the criteria for malignancy are called GT of uncertain malignant potential (GT-UMP) (3). Folpe et al. (3) proposed a classification for GT. Malignant GT should be diagnosed in the presence of atypical mitotic figures or marked atypia and mitotic activity (>5/50 high power fields [hpf]) (3). GT-UMP diagnosis is reserved in cases with superficial location and high mitotic activity (>5/50 hpf) or large size (>2 cm) and/or deep location. Our case consisted in a 2.1 cm well-defined mass in striated muscle. Microscopically it was composed of cells without mitosis or necrosis. Based on these criteria, GT-UMP was diagnosed in our patient. A minority of GT-UMP may present distant metastases, especially to the lungs (15). To mitigate metastatic risk and alleviate refractory pain, surgical excision was indicated.

MRI is thought to be the most sensitive diagnostic modality (16). GTs are described as slightly hypointense to slightly hyperintense on a T1-weighted image, and hyperintense on a T2-weighted image. Contrast enhanced T1-weighted images of the lesion demonstrate stronger enhancement (17).However, these features are not specific to glomus tumors with a specificity of 50% (18).

GTs usually present as a well-circumscribed, solid hypoechoic nodule on ultrasound, and the marked hypervascularity with arterial patterns in color Doppler imaging can be useful in the differentiation of GTs from other soft tissue tumors (19). In some cases, a “vascular stalk sign” in correlation with the presence of prominent vascular flow connecting the lesion to the adjacent soft tissue may be seen (20). However, color Doppler ultrasonography performed with two distinct ultrasound systems consistently failed to demonstrate high-intensity flow signals in this patient. The visualization of color Doppler blood flow is influenced by factors such as the depth of the mass, the angle between the ultrasound beam and blood flow, equipment performance, artifacts, and the examiner’s experience, all of which can significantly affect blood flow assessment. Therefore, the assessment of blood flow within the mass using color Doppler ultrasound has certain limitations. Contrast-enhanced ultrasound is highly sensitive in reflecting tissue and lesion perfusion, and in certain aspects, it outperforms contrast-enhanced CT in demonstrating blood supply (21). The contrast-enhanced ultrasound in this patient revealed that the majority of the mass exhibited rapid enhancement followed by rapid washout, suggesting a hyper-vascular lesion, which is consistent with the pathological characteristics of a GT. However, it is important to acknowledge that this enhancement pattern observed on CEUS is not entirely specific to GTs. Similar CEUS patterns have also been reported in certain malignant soft tissue tumors, such as liposarcoma and primary pleomorphic sarcoma (22, 23).Therefore, contrast-enhanced ultrasound is a valuable complement to conventional ultrasound, providing a more accurate assessment of the blood supply within the mass. Furthermore, the presence of non-enhancing regions within the mass may correspond to intralesional hemorrhagic liquefaction, analogous to the findings in a Giant thigh GT as reported by Pena-Burgos et al. (15) During the ultrasound-guided biopsy, the interventional physician selected tissue from the enhanced region for puncture in order to obtain more accurate biopsy results. The definitive histopathological evaluation ultimately confirmed concordance with the preoperative ultrasound-guided core needle biopsy findings. This precise histologic diagnosis corroborates the efficacy of targeting CEUS-enhanced regions during biopsy, consistent with established literature (24).

## 4 Conclusion

EGTs are extremely rare, and their atypical symptoms make direct diagnosis challenging. CEUS serves as a valuable complement to conventional imaging within a multimodal workflow, enabling precise assessment of intralesional vascularity and guiding optimal biopsy site selection. Given the nonspecific CEUS patterns in EGTs, histopathological examination is mandatory as the definitive diagnostic criterion.

## Data Availability

The original contributions presented in the study are included in the article/Supplementary Material. Further inquiries can be directed to the corresponding authors.
